# Alumni Meeting

**Published:** 1870-02

**Authors:** 


***Alumni Meeting.***

THE second annual meeting of the Alumni of Rush Medical College was held, February 2, at that institution. The meeting was called to order by the president, Dr. Alfred E. Ames, of Minneapolis, Minnesota. Dr. W. C. Hunt, secretary, read the minutes of the last meeting, which were adopted.

President Ames then delivered the annual address, which, we have only space now to say, was exceedingly able and interesting, and was listened to by a large audience with profound attention, except when interrupted by frequent applause.

The following-named officers were elected for the ensuing year:

*President* — Dr. Abner Hard, of Aurora, Illinois.
*First Vice-President* — Dr. R. C. Hamill, of Chicago.
*Second Vice-President* — Dr. V. L. Hurlbut, of Chicago.
*Treasurer* — Dr. F. A. Emmons, of Chicago.
*Secretary* — Dr. Samuel Cole, of Chicago.
*Executive Committee* — Prof. E. Ingals, chairman; Dr. Charles Parkes, Dr. S. J. Avery.

Profs. Miller and Allen, and Dr. W. C. Hunt, were appointed a committee on necrology.

The exercises were concluded by a highly successful series ot experiments, illustrative of arterial pressure, transfusion, etc., by Prof. J. W. Freer, of which we hope to have a complete report hereafter. The annual reunion was a pronounced success.

